# Radial artery-second dorsal metacarpal vein arteriovenous fistula in the first interdigital space for hemodialysis: Utilization of the most peripheral site and autologous vein in the upper limb – A case report

**DOI:** 10.1016/j.ijscr.2020.05.101

**Published:** 2020-06-11

**Authors:** Tsuyoshi Takashima, Yui Nakashima, Atsuhiko Suenaga, Masato Mizuta, Yuki Yamashita, Yuki Ikeda, Yasunori Nonaka, Makoto Fukuda, Shuichi Rikitake, Motoaki Miyazono, Kazuhisa Rikitake, Yuji Ikeda

**Affiliations:** a Department of Nephrology, National Hospital Organization, Ureshino Medical Center, 4279-3 Shimojyukukou, Uresino-machi, Ureshino, Saga, 843-0393, Japan; b Division of Nephrology, Department of Internal Medicine, Saga University Faculty of Medicine, 5-1-1 Nabeshima, Saga, Saga, 849-8501, Japan; c Department of Cardiovascular Surgery, National Hospital Organization, Ureshino Medical Center, 4279-3 Shimojyukukou, Uresino-machi, Ureshino, Saga, 843-0393, Japan

**Keywords:** Hemodialysis, Arteriovenous fistula, Radial artery, Second dorsal metacarpal vein, First interdigital space, Vascular access

## Abstract

**Introduction:**

The creation of the first arteriovenous fistula (AVF) as far distally in the upper limb as possible is ideal. We developed a new operative technique for creating a radial artery-second dorsal metacarpal vein AVF in the first interdigital space. This technique involves the creation of the AVF using the most peripheral site and autologous vein in the upper limb.

**Case presentation:**

We herein describe the steps of this technique and its successful performance in a 71-year-old man with end-stage renal disease.

**Discussion:**

This technique has several advantages including preserving many future vascular access options and providing a long segment of arterialized vein for cannulation.

**Conclusion:**

We consider this technique to be a worthwhile option and recommend the use in patients with the proper vessels for the creation of the AVF.

## Highlights

• We describe a new operative technique for creating a radial artery-second dorsal metacarpal vein AVF in the first interdigital space for hemodialysis patients.
• This technique involves the creation of the AVF using the most peripheral site and autologous vein in the upper limb.
• This technique has several advantages including preserving many future VA options and providing a long segment of arterialized vein for cannulation.
• This technique is a worthwhile option in patients with the proper vessels for the creation of the AVF.

## 1 Introduction

The native arteriovenous fistula (AVF) is cited as a better vascular access (VA) for hemodialysis than an arteriovenous graft and catheter in the guidelines [[1], [2], [3], [4], [5]]. To preserve as many future VA options as possible and provide a long segment of arterialized vein for repeated venipuncture, the guidelines also advocate creating the first AVF as far distally in the upper extremity as possible.

We previously reported an operative technique for creating a transposed radial artery-second dorsal metacarpal vein AVF (TRSDAVF) in the anatomical snuffbox (AS) using the most distal autologous vein in the upper limb [6]. We also reported an operative technique for creating a radial artery-first dorsal metacarpal vein AVF (RFDAVF) in the first interdigital space (FIS) of the dorsal hand using the most distal site in the upper limb [7].

Combining these two ideas, we developed a new operative technique for creating a radial artery-second dorsal metacarpal vein AVF (RSDAVF) in the FIS of the dorsal hand. We describe the steps of this technique and its successful performance.

This work has been reported in line with the SCARE criteria [8].

## 2 Case presentation

A 71-year-old Japanese man with end-stage renal disease due to diabetic nephropathy was emergently admitted to our hospital because of uremia with fatigue, pulmonary edema, and hyperkalemia. Therefore, hemodialysis was initiated using a temporary blood access catheter on the same day. He gradually recovered his health, and we planned to create an AVF.

**Fig. 1:**
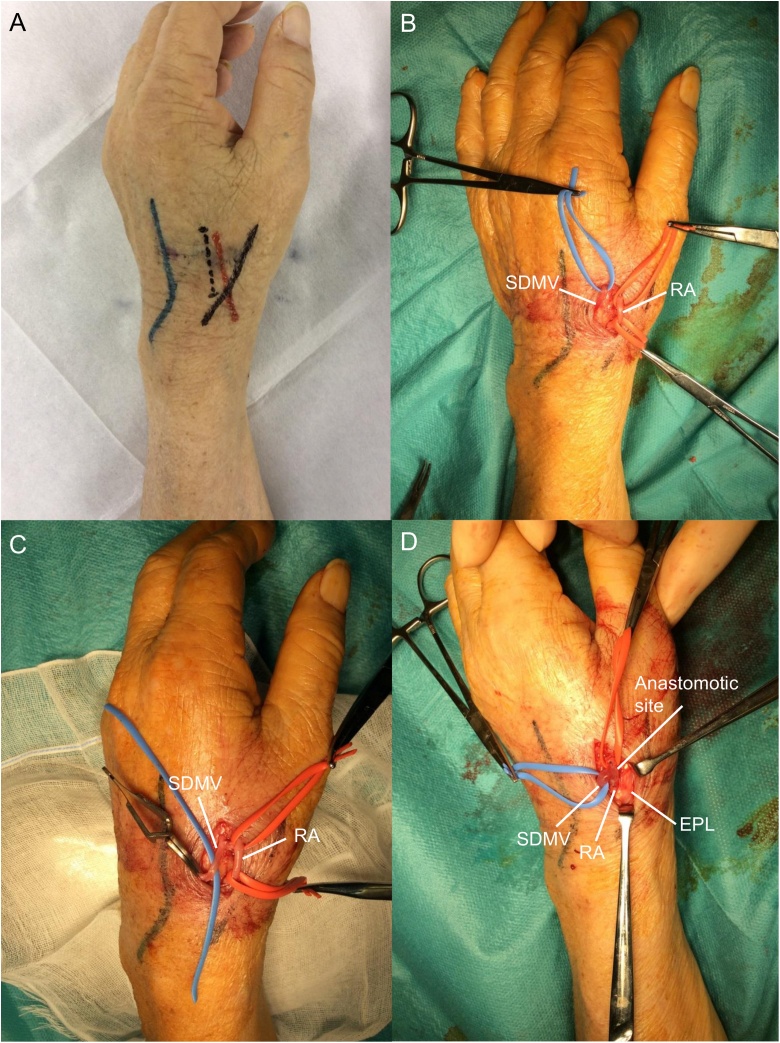
The photo illustrates the surgical technique of a radial artery-second dorsal metacarpal vein arteriovenous fistula in the first interdigital space of the dorsal hand. (A) The radial artery (red line), second dorsal metacarpal vein (blue line), tendon of the extensor pollicis longus (black line), and skin incision line (black dashed line) were preoperatively indicated using markers. (B) A longitudinal 1.5-cm skin incision was made along the slight ulnar side of the artery, and an approximately 3-cm segment of the vein and an approximately 2-cm segment of the artery were dissociated. (C) After 2000 units of heparin were administered and allowed to circulate for 5 min, the distal part of the vein was ligated and excised proximal to the ligation. (D) An end-to-side arteriovenous fistula was created between the vein and the artery. RA: radial artery, SDMV: second dorsal metacarpal vein, EPL: extensor pollicis longus.

He was right-handed. Preoperative ultrasonography of the left upper extremity showed good continuity and patency of the cutaneous vein from the wrist to the upper arm. However, the cephalic vein near the wrist was of a poor quality. Moreover, the internal diameter of the radial artery was approximately 2.0 mm in the FIS, and the second dorsal metacarpal vein (SDMV) was approximately 2.3 mm under avascularization. Therefore, we decided to create an RSDAVF in the FIS of the left dorsal hand. Thirteen days after his admission, the operation was successfully performed (Fig. 1A–D).

### 2.1 Operative technique

The radial artery, SDMV, tendonof the extensor pollicis longus, and skin incision line were preoperatively indicated using markers (Fig. 1A). The procedure was performed under local anesthesia (1% lidocaine) following a single dose of prophylactic antibiotic (cefazolin 1 g, intravenous), routine disinfection and aseptic shield.

First, a longitudinal 1.5-cm skin incision was made along the slight ulnar side of the artery over the FIS. Next, an approximately 3-cm segment of the vein was dissociated to reach and anastomose with the artery, and an approximately 2-cm segment of the artery was dissociated (Fig. 1B).

After 2000 units of heparin were administered and allowed to circulate for 5 min, the distal part of the vein was ligated and transected proximal to the ligation (Fig. 1C). The vein with visible blood reflux was flushed using a 10-ml syringe connected to a 5-Fr × 45-cm catheter, and by injecting 20–50 ml heparinized saline into the vascular lumen, we confirmed a good thrill. An end-to-side AVF whose anastomotic diameter was approximately 8 mm was then created using continuous 7/0 polypropylene sutures (Fig. 1D), and a good thrill was confirmed.

Finally, the wound was closed with 4/0 Nylon after we confirmed that no active bleeding was detected in the operative field.

**Fig. 2:**
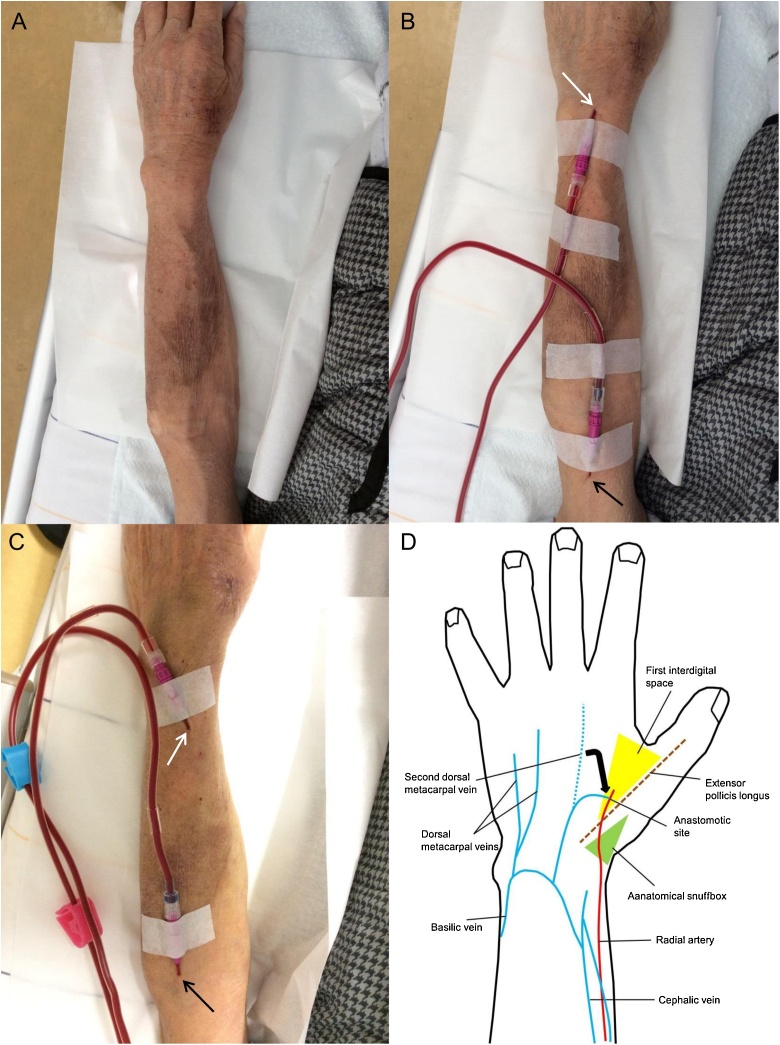
(A) A mature radial artery-second dorsal metacarpal vein arteriovenous fistula in the first interdigital space of the left dorsal hand in the present case. (B-C) Venipuncture sites during hemodialysis. Blood-removal sites: white arrows. Blood-retransfusion sites: black arrows. (D) A schematic illustration of the arteriovenous fistulas in the left dorsal hand and forearm in the present case.

The puncture of the RSDAVF was initiated 13 days after the operation. The AVF had developed sufficiently (Fig. 2A), and the blood flow rate during hemodialysis (QB) exceeded 300 mL/min. In addition, not only the superficial veins of the dorsal hand and forearm but also the cephalic and basilic veins in the forearm and upper arm had developed. He was able to receive hemodialysis without problems. The venipuncture sites are shown in Fig. 2B–C, and a schematic illustration of the AVF in the patient’s left hand and forearm is shown in Fig. 2D.

About six months later, he needed percutaneous transluminal angioplasty due to VA stenosis. It was successfully performed, and he has been on hemodialysis for seven months without additional VA interventions.

## 3 Discussion

We herein propose an RSDAVF in the FIS as another option for creating an autologous AVF using the dorsal vein of the hand. The present technique involves the creation of the AVF using the most peripheral site and most peripheral native vein in the upper extremity. To our knowledge, the present technique has not been previously reported.

**Fig. 3:**
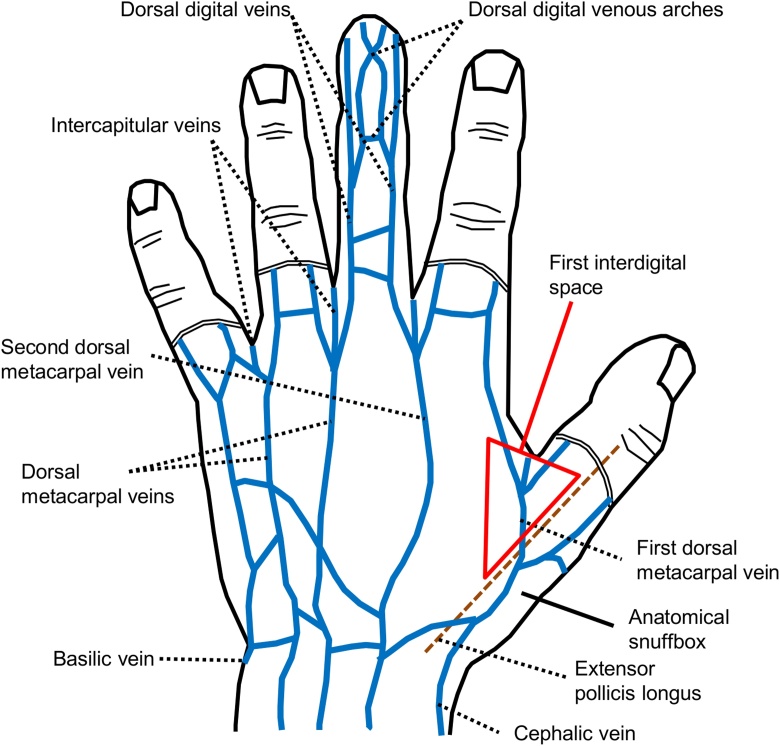
A schematic illustration of the superficial veins of the dorsum of left hand. Modified from James et al. [9].

We detailed the arterial and venous anatomy of the hand in our previous reports [6,7]. Additionally, the dorsal digital veins from the ulnar side of the index finger and from the radial side of the middle finger coalesce to form the SDMV in the second interdigital space of the dorsum of hand. Generally, the SDMV is longer and located more distally than the first dorsal metacarpal vein. A schema of the superficial veins of the dorsum of hand is shown in Fig. 3 [9].

The RSDAVF in the FIS has the following advantages: because the AVF uses the most peripheral site and autologous vein in the upper limb, it provides a long segment of vein for needling; it preserves the proximal veins for the creation of another AVF in cases of VA failure, so creation is facilitated by the presence of an already arterialized vein; the short skin incision reduces the likelihood of infection; the small caliber of the artery decreases the risk of steal phenomenon or heart failure; and the proximity between the radial artery and SDMV without transposition of the vein allows for easy anastomosis.

As described in our previous report [6], TRSDAVF in the AS needs two longitudinal skin incisions to transpose the vein, although neither wound is very large. If the distance between the artery and vein is not great, a single transverse incision may be sufficient, but when one transverse incision is used, it typically tends to be long. Furthermore, the SDMV dissociated and transposed by one transverse incision tends to be shorter than that with two longitudinal incisions. Thus, we recommend two longitudinal incisions be made to anastomose the artery and vein more easily and lengthen the segment of the arterialized vein as much as possible. Regarding one short skin incision to reduce the number and size of wounds, and make anastomosis easier to perform without the need to transpose the vein due to the proximity between the artery and vein, it is more advantageous to perform RSDAVF in the FIS, in comparing to performing TRSDAVF in the AS. As another characteristic advantage of RSDAVF in the FIS, we consider that RSDAVF in the FIS can be created as the next AVF when the TRSDAVF in the AS failed due to the venous occlusion near the anastomotic site.

Compared with an RFDAVF in the FIS, even if the cephalic vein distal to the wrist and/or the first dorsal metacarpal vein is of poor quality or thrombosed, an RSDAVF can be used to create the AVF in the FIS, as observed in the present case. Additionally, the present technique enables the superficial veins of the dorsal hand and forearm to be utilized for cannulation during hemodialysis. Furthermore, an RSDAVF can anatomically develop not only the superficial veins of the dorsal hand and forearm but also the cephalic and basilic veins in the forearm and upper arm through the venous interconnection. These characteristics provide more venipuncture sites and facilitate the creation of radial and/or ulnar proximal AVFs in the event of VA failure. Therefore, they are particularly advantageous.

Regarding the indication of an RSDAVF, we consider that the radial artery with an internal diameter of ≥2.0 mm and the SDMV under avascularization with an internal diameter of ≥2.0 mm are ideal in the light of our past experience.

## 4 Conclusion

It may be more challenging for surgeons to make the first AVF as distally in the upper extremity as possible, particularly at the dorsum of hand. Nevertheless, as described above, the creation of the AVF as distally as possible is ideal. We therefore consider these techniques using the dorsal metacarpal veins to be worthwhile options, and it is essential to select the most suitable procedure for each patient. We recommend the use of this present technique in patients with the proper SDMV and radial artery in the FIS for the creation of the AVF.

## Declaration of Competing Interest

The authors declare no conflict of interest.

## Funding

This research did not receive any specific grant(s) from funding agencies in the public, commercial, or not-for-profit sectors.

## Ethical approval

No Institutional Review Board is required for publication of a case report at our institution.

## Consent

Informed consent was obtained from the patient for publication of this case report and any accompanying images.

## Author contribution

Takashima T considered the operative technique, and Takashima T and Nakashima Y performed the surgery.

Takashima T, Suenaga A, Mizuta M, Yamashita Y, Ikeda Yuki, Nonaka Y, Fukuda M, Rikitake S, Miyazono M and Rikitake K contributed in the collection of literature.

Takashima T made drafted and edited the manuscript.

Ikeda Yuji gave final approval of the manuscript.

## Registration of research studies

The paper is not a research study.

## Guarantor

Takashima T.

## Provenance and peer review

Not commissioned, externally peer-reviewed.
